# Outcomes and predictors of in-hospital mortality among patients admitted to the intensive care or step-down unit after a rapid response team activation: A retrospective cohort study

**DOI:** 10.1371/journal.pone.0317429

**Published:** 2025-04-28

**Authors:** Vinicius Barbosa Galindo, Thais Dias Midega, Guilherme Martins de Souza, Fábio Barlem Hohmann, Mayara Laise Assis, Ricardo Luiz Cordioli, Roseny dos Reis Rodrigues, Gustavo Faissol Janot de Matos, Andréia Pardini, Michele Jaures, Bruno de Arruda Bravim, Claudia Regina Laselva, Constantino Jose Fernandes Jr, Thiago Domingos Corrêa

**Affiliations:** Intensive Care Unit – Hospital Israelita Albert Einstein, São Paulo, Brazil; King Saud University College of Medicine, SAUDI ARABIA

## Abstract

**Introduction:**

It has been demonstrated that the implementation of rapid response teams (RRT) may improve clinical outcomes. Nevertheless, predictors of mortality among patients admitted to the intensive care unit (ICU) or to the step-down unit (SDU) after a RRT activation are not fully understood.

**Objective:**

To describe clinical characteristics, resource use, main outcomes, and to address predictors of in-hospital mortality among patients admitted to the ICU/SDU after RRT activation.

**Methods:**

Retrospective single-center cohort study conducted in a medical-surgical ICU/SDU located in a private quaternary care hospital. Adult patients admitted to the ICU or SDU between 2012 and 2020 were compared according to in-hospital mortality. A multivariate logistic regression analysis was performed to identify independent predictors of in-hospital mortality.

**Results:**

Among the 3841 patients included in this analysis [3165 (82.4%) survivors and 676 (17.6%) non-survivors], 1972 (51.3%) were admitted to the ICU and 1869 (48.7%) were admitted to the SDU. Compared to survivors, non-survivors were older [76 (64–87) yrs. vs. 67 (50–81) yrs.; p < 0.001], had a higher SAPS 3 score [64 (56–72) vs. 49 (40–57); p < 0.001], and had a longer length of stay (LOS) before unit admission [8 (3–19) days vs. 2 (1–7) days; p < 0.001). Non-survivors used more non-invasive ventilation (NIV) (42.2% vs. 20.9%; p < 0.001), mechanical ventilation (MV) (36.7% vs. 9.3%; p < 0.001), vasopressors (39.2% vs. 12.3%; p < 0.001), renal replacement therapy (15.5% vs. 4.3%; p < 0.001), and blood components transfusion (34.9% vs. 14.0%; p < 0.001). Independent predictors of in-hospital mortality were the SAPS 3 score, the Charlson Comorbidity Index, LOS before unit admission, immunosuppression, respiratory rate < 8 or > 28 ipm criteria for RRT activation, RRT activation during the night shift, and the need for high-flow nasal cannula, NIV, MV, vasopressors, and blood components transfusion.

**Conclusion:**

Multiple factors may affect outcomes of ICU/SDU-admitted patients after RRT activation. Therefore, efforts should be made to boost RRT effectiveness to improve patient safety.

## Introduction

Failure to promptly recognize and address clinical deterioration stands as a primary contributor to harm among hospitalized patients [1–3]. The endorsement of Rapid Response Teams (RRT), also known as Medical Emergency Teams (MET), aims to decrease harm and improve safety for hospitalized patients, and has been supported by the Institute of Healthcare Improvement (IHI) [4] as well as by various medical specialty societies [5,6]. The RRT is, by definition, a multidisciplinary emergency team composed of trained intensive care unit (ICU) physicians, nurses, and respiratory therapists who provide specialized care to patients presenting clinical deterioration outside the ICU [4–6].

It has been demonstrated that the implementation of RRT may improve inpatient care outcomes by providing a proactive and timely response to clinical deterioration, thereby mitigating unsafe ICU transfers, the development of cardiac arrest, and death [7–9]. Furthermore, several safety mechanisms and tools have been developed and incorporated into healthcare systems to further improve inpatient security and clinical outcomes [10–15]. For instance, the use of early warning score systems such as the National Early Warning Score (NEWS) [11] and the Modified Early Warning Score (MEWS) [10] allow an early identification of clinical deterioration in ward patients, triggering prompt and timely interventions to prevent the occurrence of deleterious events. Moreover, safety huddles [12,14], telemetry monitoring [13], and the identification of patients at a higher risk of clinical deterioration outside the ICU, commonly referred to as “watchers” [15,16], have demonstrated promising results in enhancing safety awareness among multidisciplinary care teams [17].

Nevertheless, predictors of in-hospital mortality among patients admitted to the ICU or to the step-down unit (SDU) following RRT activation are not completely understood. We postulated that patient-related factors [i.e., age, comorbidities, frailty, reason for hospitalization, and length of hospital stay (LOS) before RRT activation] and RRT activation factors (i.e., trigger, time span between clinical deterioration, and activation and response time) may be associated with in-hospital mortality in patients admitted to the ICU/SDU after RRT activation. Therefore, our primary objective was to address the predictors of in-hospital mortality among this patient cohort. Secondary objectives included describing clinical characteristics, resource use, and main outcomes.

## Materials and methods

### Study design

Retrospective, single-center cohort study. This study was approved by the local Ethics Committee at Hospital Israelita Albert Einstein (CAAE: 40072720.0.0000.0071), and the need for informed consent was waived. This study was reported in accordance with the Strengthening the Reporting of Observational Studies in Epidemiology (STROBE) statement [18].

### Setting

This study was conducted in a private quaternary care hospital located in São Paulo, Brazil. The Hospital Israelita Albert Einstein has 724 inpatient beds. Of these, 54 were open medical-surgical adult ICU beds, and 95 were adult SDU beds. The SDU serves patients considered too unstable to be admitted to the wards but without requiring the full availability of resources in an ICU, and patients discharged from the ICU recovering from a critical condition.

### Patients

All adult (≥18 years) patients admitted to ICU or SDU after RRT activation between January 1, 2012, and December 31, 2020, were eligible for this study. The exclusion criteria comprise pediatric patients (<18 years), admissions after blue code activation (unexpected cardiac or respiratory arrest), and ICU/SDU readmissions. In the case of recurrent RRT activation and ICU/SDU admission for the same patient, only the first ICU/SDU admission was included in this analysis. Patients under end-of-life care or no escalation of treatment order are usually not eligible for RRT activation. Therefore, these patients were not included in this analysis.

### Data collection and study variables

All study data were retrieved from an institutional yellow code data bank, and from Epimed Monitor System® (Epimed Solutions, Rio de Janeiro, Brazil) [19], which are structured electronic case report forms where patients’ data are prospectively entered by trained hospital case managers. All data were extracted by an independent research assistant who did not participate in this study. All data were fully anonymized before being made available to the researchers. The data were accessed and extracted on 28/06/2021.

The collected variables included demographics, comorbidities, Simpliﬁed Acute Physiology Score (SAPS 3 score) [20], Sequential Organ Failure Assessment (SOFA) score [21] at ICU/SDU admission, Charlson Comorbidity Index (CCI) [22], Modified Frailty Index (MFI) [23], reason for ICU or SDU admission, the MEWS [10] at the moment of RRT activation, reason for RRT activation, patient’s location before unit admission, the time elapsed between patient deterioration and RRT activation, the time between RRT activation and the team’s arrival, destination after RRT activation (ICU or SDU), resource use and organ support [vasopressors, non-invasive ventilation (NIV), high flow nasal cannula (HFNC), mechanical ventilation (MV), renal replacement therapy (RRT) and blood components transfusion] during the first hour of ICU/SDU admission and during the ICU/SDU stay, unit and hospital length of stay (LOS), and hospital mortality.

### Rapid response team

The intensivist-led RRT was implemented in our institution in 2008. The RRT is headed by a senior staff member, who is available 24 hours a day and has expertise in critical patient care, airway management, and advanced cardiovascular life support.

Criteria to activate the RRT included at least one of the following: peripheral oxygen saturation (SpO_2_) < 90%, respiratory rate < 8 or > 28 incursions per minute (ipm), systolic blood pressure (SBP) < 90 mmHg or SBP > 180 mmHg, heart rate < 40 or > 130 beats per minute (bpm), altered neurological status, seizures, MEWS > 6 points, and a multidisciplinary staff member expressing significant concern about the patient condition (“worried” staff). The RRT is activated via a dedicated single emergency telephone number (Yellow Code). Once activated, the RRT was expected to assess the patient within five minutes and define the patient’s subsequent allocation based on clinical judgment and institutional protocols. Additionally, unexpected cardiac or respiratory arrest on the wards is managed by a specialized team known as the Blue Code team. This team, led by a senior certified cardiologist in advanced cardiac life support, is also available 24 hours a day.

### Statistical analysis

Categorical variables were reported as absolute and relative frequencies, while continuous variables were presented as median with interquartile ranges (IQR). Normality was evaluated using the Kolmogorov-Smirnov test.

Comparisons were performed between survival and non-survival patients. Categorical variables were compared with the X^2^ test or Fisher’s exact test as appropriate. Continuous variables were compared using an independent *t*-test or the Mann–Whitney U test in cases of non-normal distribution.

Univariable logistic regression analysis was performed to identify which predictors were associated with in-hospital mortality. Multivariable logistic regression analyses with a backward elimination procedure, including all the predictors showing a p-value < 0.20 in the univariable analysis, were undertaken to obtain an adjusted odds ratio (OR) along with 95% confidence interval (CI) and to identify which predictors were independently associated with in-hospital mortality. Therefore, the final model contained only variables significantly associated with in-hospital mortality after a multivariable backward logistic regression analysis.

To avoid collinearity, the characteristics included in the SAPS 3 score (namely, age) and the comorbidities included in the CCI [namely, diabetes mellitus, chronic heart failure (CHF), chronic kidney disease (CKD), solid tumor, liver cirrhosis, hematological malignancy, and severe chronic obstructive pulmonary disease (COPD)] were not individually added to the model. Collinearity was assessed using the variance inflation factor (VIF). A VIF > 2.5 was arbitrarily defined as an indicator of collinearity.

We tested the linearity assumption for continuous variables included in logistic regression models by analyzing the interaction between each predictor and its own log (natural log transformation) [24]. Whenever the linearity assumption was violated, continuous numerical variables were categorized [24]. Final multivariable logistic regression model discrimination [area under a receiver operating characteristic curve (AUC)] and calibration (Hosmer-Lemeshow chi-square statistic) were reported [25].

Two-tailed tests were used, and statistical significance was set at p < 0.05. All analyses were performed using the IBM Statistical Package for the Social Sciences (SPSS) Statistics for Macintosh, version 28 (IBM Corp., Armonk, NY, USA).

## Results

### Patients

**Fig 1:**
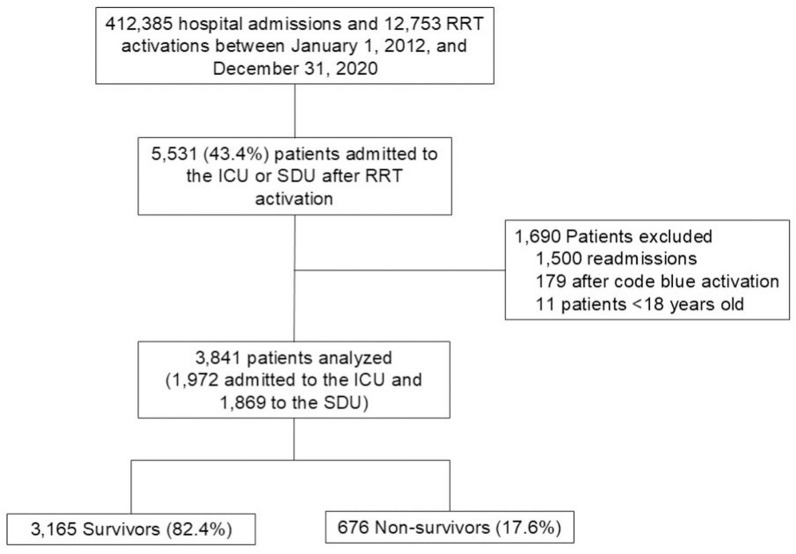
Flowchart of patients admitted to the intensive care unit or step-down unit after rapid response team activation. ICU: Intensive Care Unit; SDU: Step-down Unit; RRT: Rapid Response Team.

Between January 1, 2012, and December 31, 2020, there were 412,385 hospital admissions and 12,753 RRT activations. Of these, 5,531 (43.4%) resulted in ICU or SDU admissions after RRT activation. After the exclusion of 1,690 ICU/SDU admissions due to ICU/SDU readmissions after RRT activation, admissions after code blue activation, and age below 18 years, 3,841 patients were included in the final analysis. Among these, 3,165 (82.4%) patients were survivors, while 676 (17.6%) were non-survivors (Fig 1). Among the non-survivors, 34.4% (232/676 patients) died during the ICU/SDU stay.

The baseline characteristics of the studied patients are presented in Table 1. Compared to survivors, non-survivors were older [76 (64–87) vs. 67 (50–81) years; p < 0.001], had a higher proportion of males (55.5% vs. 49.7%; p = 0.007), a higher SAPS 3 score [64 (56–72) vs. 49 (40–57) points; p < 0.001], a higher SOFA score [4 (2–7) vs. 1 (0–3) points; p < 0.001], were more frequently frail (21.6% vs. 11.5%; p < 0.001), had a higher CCI [3 (2–6) vs. 1 (0–3) points; p < 0.001], a longer LOS before unit admission [8 (3–19) vs. 2 (1–7) days; p < 0.001], exhibited a higher prevalence of solid tumors (44.4% vs. 25.6%; p < 0.001), immunosuppression (35.9% vs. 16.5%; p < 0.001), chronic heart failure (18.5% vs. 12.3%; p < 0.001), hematological malignancy (14.3% vs. 5.7%; p < 0.001) and chronic kidney disease (14.1% vs. 7.9%; p < 0.001) (Table 1).

**Table 1:** Baseline characteristics of studied patients.

Characteristics	All patients 3841/3841 (100.0%)	Survivors 3165/3481 (82.4%)	Non-survivors 676/3841 (17.6%)	p-value
Age, years (median, IQR)	70 (52-83)	67 (50-81)	76 (64-87)	<0.001^*^
Sex (male), n (%)	1948/3841 (50.7)	1573/3165 (49.7)	375/676 (55.5)	0.007^†^
SAPS 3 score^‡^ (median, IQR)	51 (42-60)	49 (40-57)	64 (56-72)	<0.001^*^
SOFA score^§^ (median, IQR)	2 (0-4)	1 (0-3)	4 (2-7)	<0.001^*^
MFI^¶^ points (median, IQR)	2 (1-3)	2 (1-3)	2 (1-3)	<0.001^*^
MFI^¶^ frail patient, n (%)	511/3841 (13.3)	365/3165 (11.5)	146/676 (21.6)	<0.001^†^
CCI^µ^ (median, IQR)	2 (0-3)	1 (0-3)	3 (2-6)	<0.001^*^
MEWS^ʎ^ (median, IQR)	2 (1-3)	2 (1-3)	2 (1-4)	<0.001^*^
LOS before unit admission (median, IQR)	3 (1-9)	2 (1-7)	8 (3-19)	<0.001^*^
Reason for ICU admission, n (%)				
Nonoperative	3426/3841 (89.2)	2776/3165 (87.7)	650/676 (96.2)	<0.001^†^
Elective Surgery	276/3841 (7.2)	263/3165 (8.3)	13/676 (1.9)	
Emergency/Urgent surgery	139/3841 (3.6)	126/3165 (4.0)	13/676 (1.9)	
Admission diagnoses^g^, n (%)				<0.001^†^
Nonoperative	3426/3841 (89.2)	2776/3165 (87.7)	650/676 (96.2)	
Cardiovascular	891/3841 (23.2)	788/3165 (24.9)	103/676 (15.2)	
Respiratory	889/3841 (23.1)	653/3165 (20.6)	236/676 (34.9)	
Gastrointestinal	202/3841 (5.3)	157/3165 (5.0)	45/676 (6.7)	
Neurologic	404/3841 (10.5)	344/3165 (10.9)	60/676 (8.9)	
Sepsis	812/3841 (21.1)	637/3165 (20.1)	175/676 (25.9)	
Trauma	26/3841 (0.7)	21/3165 (0.7)	5/676 (0.7)	
Metabolic	37/3841 (1.0)	31/3165 (1.0)	6/676 (0.9)	
Hematologic	25/3841 (0.7)	19/3165 (0.6)	6/676 (0.9)	
Renal diseases	17/3841 (0.4)	12/3165 (0.4)	5/676 (0.7)	
Other causes	123/3841 (3.2)	114/3165 (3.6)	9/676 (1.3)	
Postoperative	415/3841 (10.8)	389/3165 (12.3)	26/676 (3.8)	
Vascular/Cardiovascular	76/3841 (2.0)	69/3165 (2.2)	7/676 (1.0)	
Respiratory	13/3841 (0.3)	12/3165 (0.4)	1/676 (0.1)	
Gastrointestinal	125/3841 (3.3)	116/3165 (3.7)	9/676 (1.3)	
Neurologic	15/3841 (0.4)	11/3165 (0.3)	4/676 (0.6)	
Renal	41/3841 (1.1)	40/3165 (1.3)	1/676 (0.1)	
Gynecologic	7/3841 (0.2)	7/3165 (0.2)	0/676 (0.0)	
Orthopedic	31/3841 (0.8)	30/3165 (0.9)	1/676 (0.1)	
Other causes	107/3841 (2.8)	104/3165 (3.3)	3/676 (0.4)	
Comorbidities, n (%)				
Systemic hypertension	1843/3355 (54.9)	1502/2686 (55.9)	341/669 (51.0)	0.024^†^
Diabetes mellitus	1075/3355 (32.0)	857/2686 (31.9)	218/669 (32.6)	0.771^†^
CHF NYHA Class 2,3 or 4^β^	454/3355 (13.5)	330/2686 (12.3)	124/669 (18.5)	<0.001^†^
CKD	307/3355 (9.2)	213/2686 (7.9)	94/669 (14.1)	<0.001^†^
CKD requiring long-term dialysis	203/3355 (6.1)	147/2686 (5.5)	56/669 (8.4)	0.006^†^
Solid tumor	984/3355 (29.3)	687/2686 (25.6)	297/669 (44.4)	<0.001^†^
Liver cirrhosis	120/3355 (3.6)	84/2686 (3.1)	36/669 (5.4)	0.007^†^
Hematological malignancy	250/3355 (7.5)	154/2686 (5.7)	96/669 (14.3)	<0.001^†^
Immunosuppression	684/3355 (20.4)	444/2686 (16.5)	240/669 (35.9)	<0.001^†^
Severe COPD	334/3355 (10.0)	260/2686 (9.7)	74/669 (11.1)	0.319^†^

Data presented as median (interquartile range) or n/total n (%).

* p values were calculated with the of Mann-Whitney U test.

† P values were calculated with the use of chi-square test.

SAPS 3 =  Simplified Acute Physiology Score 3; SOFA =  Sequential Organ Failure Assessment score; MFI =  Modified frailty index; MEWS: Modified Early Warning Score; LOS =  Length of stay; CFH NYHA Class =  Chronic Heart Failure, New York Heart Association functional classification; CKD =  Chronic Kidney Disease; COPD: Chronic obstructive pulmonary disease.

‡ Scores on SAPS 3 range from 0 to 217, with higher scores indicating more severe illness and higher risk of death.

§ SOFA score quantify organ dysfunction in 6 organ systems, resulting in an aggregated score that ranges from 0 to 24, with higher scores indicating greater dysfunction and greater risk of mortality. Data available for 892 patients.

¶ MFI is comorbidity-based risk stratification tool, based on a 5-factor index, designed to stratify patients based on the level of frailty and predict mortality and morbidity.

µ Charlson Comorbidity Index predicts 10-year survival in patients with multiple comorbidities, with score of zero indicating that no comorbidities were found. The higher the score, the more likely the predicted outcome will result in mortality or higher resource use.

ʎ MEWS is a score to detect early clinical deterioration, including systolic blood pressure, heart rate, respiratory rate, temperature, mental status, and urine output. Values ≥5 points indicates increased likelihood of death or admission to an intensive care unit. Data available for 926 patients.

^g^List of diagnosis based on the APACHE III disease category; proportions may not add to 100% due to rounding.

β NYHA classification for heart failure is based on the severity of the symptoms, it ranges from I to IV, and Class IV indicates that the patient is unable to carry on any physical activity without discomfort, symptoms of heart failure in rest, and if any physical activity is undertaken, discomfort increases.

### RRT activation characteristics

Most RRT activations occurred on the ward (93.6%) and approximately half of them during the night shift (Table 2). The main reason for RRT activation was a multidisciplinary staff member “worried” about the patient’s condition (64.2%), followed by SpO_2_ < 90% (30.8%) and SBP < 90 mmHg (20.4%). Notably, only 22.2% of activations were based solely on the subjective ‘worried’ criterion. The median (IQR) time between patient deterioration and RRT activation and between RRT activation and team arrival was, respectively, 3 (2–6) minutes and 3 (2–5) minutes. Out of the 3,841 patients attended by RRT, 1,972 (51.3%) were transferred to the ICU, and 1,869 (48.7%) were transferred to the SDU (Table 2).

**Table 2:** Rapid response team activation characteristics.

Characteristics	All patients 3841/3841(100.0%)	Survivors 3165/3841(82.4%)	Non-survivors 676/3841 (17.6%)	p-value
Location of RRT activation^#^, n (%)				0.004†
Ward	3158/3375 (93.6)	2584/2761 (93.6)	574/614 (93.5)	
Surgical/Obstetric center	63/3375 (1.9)	59/2761 (2.1)	4/614 (0.7)	
Hemodialysis center	26/3375 (0.8)	16/2761 (0.6)	10/614 (1.6)	
Other	128/3375 (3.8)	102/2761 (3.7)	26/614 (4.2)	
Reason for RRT activation^&^, n (%)				
Staff member worried about patient’s condition	2464/3839 (64.2)	2050/3163 (64.8)	414/676 (61.2)	0.085†
SpO_2_ < 90%	1184/3839 (30.8)	886/3163 (28.0)	298/676 (44.1)	<0.001†
SBP < 90 mmHg	782/3839 (20.4)	625/3163 (19.8)	157/676 (23.2)	0.043†
Heart rate < 40 or > 130 bpm	765/3835 (19.9)	638/3160 (20.2)	127/675 (18.8)	0.448†
Respiratory rate < 8 or > 28 ipm	624/3839 (16.3)	439/3163 (13.9)	185/676 (27.4)	<0.001†
Altered neurological status	550/3839 (14.3)	399/3163 (12.6)	151/676 (22.3)	<0.001†
SBP > 180 mmHg	254/3838 (6.6)	222/3163 (7.0)	32/675 (4.7)	0.033†
Seizures	150/3839 (3.9)	127/3163 (4.0)	23/676 (3.4)	0.524†
MEWS^§^ > 6	9/1109 (0.8)	5/942 (0.5)	4/167 (2.4)	0.045†
Night Shift, n (%)	2050/3841 (53.4)	1663/3165 (52.5)	387/676 (57.2)	0.029†
Time (min) between patient deterioration and RRT activation, (median, IQR)	3 (2-6)	3 (2-6)	4 (2-7)	0.075^*^
Time (min) between RRT activation and team arrival, (median, IQR)	3 (2-5)	3 (2-5)	3 (2-4)	0.048^*^
Destination after RRT activation, n (%)				<0.001†
ICU	1972/3841 (51.3)	1507/3165 (47.6)	465/676 (68.8)	
SDU	1869/3841 (48.7)	1658/3165 (52.4)	211/676 (31.2)	

Data presented as median (interquartile range) or n/total n (%).

* † P values were calculated with the use of chi-square test or with Mann-Whitney U test.

RRT =  Rapid response team; SpO_2_ =  Peripheral oxygen saturation; SBP =  systolic blood pressure; MEWS =  Modified Early Warning Score; ICU =  intensive care unit; SDU =  Step-down unit.

# Other: Outpatient clinic, blood bank, rehabilitation center, radiotherapy, radiology center.

& : Proportions may not add to 100% as patients can have more than one criterion for RRT activation.

§ MEWS is a score to detect early clinical deterioration, including systolic blood pressure, heart rate, respiratory rate, temperature, mental status, and urine output. Values ≥5 points indicates increased likelihood of death or admission to an intensive care unit. Data available for 926 patients.

Compared to survivors, non-survivors had a higher frequency of RRT activation triggered by SpO_2_ < 90% (44.1% vs. 28.0%; p < 0.001), respiratory rate < 8 ipm or > 28 ipm (27.4% vs. 13.9%; p < 0.001), SBP < 90 mmHg (23.2% vs. 19.8%; p = 0.043) and altered neurological status (22.3% vs. 12.6%; p < 0.001), and were more commonly transferred to the ICU (68.8% vs. 47.6%; p < 0.001) (Table 2).

### Resource use and outcomes

Whitin the first hour of ICU/SDU admission, non-survivors used NIV (23.2% vs. 11.0%; p < 0.001), MV (12.0% vs. 3.9%; p < 0.001), and vasopressors (15.8% vs. 5.0%; p < 0.001) more frequently compared to survivors (Table 3). The utilization of supportive therapies during the entire ICU/SDU stay was also higher among non-survivors: NIV (42.2% vs. 20.9%; p < 0.001), MV (36.7% vs. 9.3%; p < 0.001), vasopressors (39.2% vs. 12.3%; p < 0.001), renal replacement therapy (15.5% vs. 4.3%; p < 0.001), and blood components transfusion (34.9% vs. 14.0%; p < 0.001) (Table 3). Additionally, non-survivors had a more prolonged unit [3 (1–8) vs. 2 (1–4) days; p < 0.001] and hospital [27 (14–58) vs. 11 (5–24) days; p < 0.001] LOS compared to survivors (Table 3).

**Table 3:** Resource use and study outcomes.

Characteristics	All patients 3841/3841 (100.0%)	Survivors 3165/3481 (82.4%)	Non-survivors 676/3841 (17.6%)	p-value
Support during first hour after unit admission, n (%)				
Non-invasive ventilation	506/3841 (13.2)	349/3165 (11.0)	157/676 (23.2)	<0.001†
Vasopressors	266/3841 (6.9)	159/3165 (5.0)	107/676 (15.8)	<0.001†
Mechanical ventilation	204/3841 (5.3)	123/3165 (3.9)	81/676 (12.0)	<0.001†
Renal replacement therapy	12/3841 (0.3)	8/3165 (0.3)	4/676 (0.6)	0.292†
Support during unit stay, n (%)				
Non-invasive ventilation	945/3841 (24.6)	660/3165 (20.9)	285/676 (42.2)	< 0.001†
Blood component transfusion	679/3841 (17.7)	443/3165 (14.0)	236/676 (34.9)	<0.001†
Vasopressors	653/3841 (17.0)	388/3165 (12.3)	265/676 (39.2)	<0.001†
Mechanical ventilation	541/3841 (14.1)	293/3165 (9.3)	248/676 (36.7)	<0.001†
Renal replacement therapy	240/3841 (6.2)	135/3165 (4.3)	105/676 (15.5)	<0.001†
High-flow nasal cannula	73/3841 (1.9)	52/3165 (1.6)	21/676 (3.1)	0.018†
Unit LOS, days (median, IQR)	2 (1-4)	2 (1-4)	3 (1-8)	<0.001 *
Hospital LOS, days (median, IQR)	13 (6-29)	11 (5-24)	27 (14-58)	<0.001 *

Data presented as median (interquartile range) or n/total n (%).

*† P values were calculated with the use of chi-square test or with Mann-Whitney U test.

ICU = intensive care unit; LOS = length of stay.

### Predictors of hospital mortality

The results of univariable and multivariate logistic regression analysis are presented in Table 4. Independent predictors of increased in-hospital mortality on the multivariate analysis included the SAPS 3 score, the CCI, LOS before unit admission, presence of immunosuppression, respiratory rate < 8 or > 28 ipm criteria for RRT activation, RRT activation during the night shift, and the need for HFNC, NIV, MV, vasopressors, and blood components transfusion during the ICU/SDU stay (Table 4).

**Table 4:** Univariate and multivariate logistic regression analysis addressing predictors of in-hospital mortality in patients admitted to the intensive care unit or step-down unit after rapid response team activation.

	Univariable Analysis		Multivariable Analysis	
Predictors	OR (CI 95%)	p-value	OR (CI 95%)	p-value
Sex (male)	1.26 (1.07-1.49)	0.006		
SAPS 3 score^*^				
≤ 42	1.00 (Reference)		1.00 (Reference)	
43-50	4.06 (2.46-6.72)	<0.001	1.81 (1.07-3.07)	0.027
51-59	9.10 (5.71-14.49)	<0.001	3.00 (1.83-4.92)	<0.001
≥ 60	35.71 (22.78-55.96)	<0.001	6.15 (3.75-10.08)	<0.001
MFI frail patient	2.11 (1.71-2.62)	<0.001		
Charlson Comorbidity Index^*^				
0	1.00 (Reference)		1.00 (Reference)	
1-2	4.34 (3.18-5.93)	<0.001	2.05 (1.41-3.00)	<0.001
3-4	7.08 (5.10-9.83)	<0.001	2.02 (1.35-3.03)	<0.001
≥ 5	17.53 (12.64-24.31)	<0.001	4.51 (2.98-6.83)	<0.001
LOS before unit admission^*^				
≤ 1	1.00 (Reference)		1.00 (Reference)	
2-3	1.50 (1.11-2.02)	0.008	1.26 (0.90-1.78)	0.181
4-8	3.08 (2.36-4.02)	<0.001	2.14 (1.57-2.91)	<0.001
≥ 9	6.00 (4.74-7.60)	<0.001	2.49 (1.88-3.29)	<0.001
Illness Category				
Nonoperative	1 (Reference)			
Elective Surgery	0.21 (0.12-0.37)	<0.001		
Urgency/ Emergency surgery	0.44 (0.25- 0.79)	0.005		
Underlying disease				
Systemic hypertension	0.82 (0.69-0.97)	0.021	0.77 (0.63-0.94)	0.012
Immunosuppression	2.83 (2.34-3.41)	<0.001	1.54 (1.22-1.95)	<0.001
Reason for RRT activation				
SpO_2_ < 90%	2.03 (1.71-2.40)	<0.001		
Respiratory rate < 8 or > 28 ipm	2.34 (1.92- 2.85)	<0.001	1.51 (1.18-1.93)	<0.001
SBP < 90 mmHg	1.23 (1.01-1.50)	0.043		
SBP > 180 mmHg	0.66 (0.45-0.96)	0.032		
Altered neurological status	1.99 (1.62-2.46)	<0.001		
Staff member worried	0.86 (0.72-1.02)	0.077		
Night Shift	1.21 (1.02-1.43)	0.026	1.35 (1.11-1.66)	0.003
Time (min) between patient deterioration and RRT activation	1.00 (0.99-1.00)	0.246		
Time (min) between RRT activation and team arrival^*^				
< 2	1 (Reference)			
2-3	1.06 (0.74-1.53)	0.740		
4-5	0.94 (0.65-1.36)	0.74		
≥ 6	0.61 (0.37-1.01)	0.056		
Support during ICU/SDU stay				
High-flow nasal canula	1.92 (1.15-3.21)	0.013	2.28 (1.18-4.37)	0.014
Non-invasive ventilation	2.77 (2.32-3.30)	<0.001	1.49 (1.20-1.86)	<0.001
Mechanical ventilation	5.68 (4.66-6.92)	<0.001	2.56 (1.97-3.40)	<0.001
Vasopressors	4.62 (3.83-5.57)	<0.001	1.68 (1.30-2.17)	<0.001
Renal replacement therapy	4.13 (3.15-5.41)	<0.001		
Blood component transfusion	3.30 (2.73-3.98)	<0.001	1.56 (1.23-1.97)	<0.001

OR: odds ratio; 95%CI: 95% confidence interval; SAPS 3: Simplified Acute Physiology Score 3; MFI: modified frailty index; LOS =  Length of stay (days); SpO_2_ =  Peripheral oxygen saturation; SBP =  Systolic blood pressure.

* Variables categorized according to percentiles since linearity assumption was violated.

The multivariable model (n = 3350 patients) had an area under the Receiver Operating Characteristic curve (95%CI) of 0.85 (0.83–0.86) and a Hosmer-Lemeshow χ2 of 19,867 (p = 0.011).

## Discussion

In this single-center retrospective cohort study, we found that patients admitted to the ICU/SDU due to clinical deterioration and RRT activation often required organ support therapies, mainly NIV, vasopressors, and blood transfusion. Furthermore, approximately one in five patients who had RRT activation and who were admitted to the ICU/SDU did not survive to hospital discharge. Additionally, there were several predictors of increased mortality among patients with clinical instability attended by RRT.

Hospitalized patients attended by RRT are commonly characterized by advanced age, the presence of multiple comorbidities and frailty, which have been associated with an increased risk of poor clinical outcomes [2,26–30]. Moreover, patients who had RRT activation often required bedside interventions and organ support therapies [26,31,32]. Goh and cols demonstrated that approximately 80% of patients who required RRT activation needed at least one intervention, mainly ICU/SDU admission, point-of-care ultrasound, fluid administration, vasopressors, and endotracheal intubation [32]. Therefore, the adoption of effective safety protocols and proactive interventions that allow an early recognition and prompt assessment of patients with clinical instability outside the ICU/SDU has been recommended [4,6,33,34].

Triggers for RRT activation vary widely among different institutions [28,29,35]. Similarly to our findings, Shappell and cols., using a large multicenter registry of patients attended by RRT across the United States, demonstrated that SBP, hospital LOS before RRT call, and respiratory rate were important predictors of in-hospital mortality [29]. Nevertheless, it has been demonstrated that RRT activation based on subjective criteria, i.e., a multidisciplinary staff concern about patients’ clinical condition in the absence of a specific physiologic trigger (also known as “worried” staff or “nursing concern”) represents an important tool that allows the multidisciplinary team to escalate care aiming to prevent adverse events in hospitalized patients [36]. We found that approximately two-thirds of all RRT activations were triggered by the “worried” criteria in association with any other physiologic criteria, and approximately one-fifth of RRT were activated exclusively by “worried” staff criteria. Similar results were reported by other authors in a cluster randomized trial conducted in 23 hospitals addressing the characteristics and frequency of triggers for emergency team activation [37].

We demonstrated that the patient’s comorbidity (CCI and immunosuppression) and severity (SAPS 3 score), LOS before unit admission, respiratory depression or tachypnea, RRT activation during the night shift, and the need for supportive therapy during the unit stay were independent predictors of in-hospital death among patients admitted to the ICU/SDU after RRT activation. Although the SAPS 3 score was originally developed to predict mortality upon ICU admission [20], our findings suggest that it may also serve as a marker of poor outcomes in patients admitted to the ICU/SDU after a RRT activation. In addition, comorbidities play a significant role in the outcomes of patients requiring RRT activation. For example, patients with hematological malignancies who require RRT activation face a higher risk of adverse outcomes [38]. The participation of RRT in these instances can facilitate prompt identification of clinical deterioration, which has been associated with faster ICU admissions and reduced SOFA scores during the ICU stays [39].

Moreover, it has been demonstrated that a prolonged LOS prior to the RRT activation [29,40–43], code activation during the night shift [28,44], and the need for life support therapies [41,43,45] are important risk factors for in-hospital death among patients receiving RRT calls. For instance, Calzavacca and cols. identified vasopressor use as a predictor of in-hospital mortality in patients receiving multiple RRT activations [41], while Lee and cols demonstrated that the duration of hospitalization before RRT activation, and the need for endotracheal intubation were independent predictors of in-hospital mortality [43]. Additionally, the presence of respiratory failure at the time of RRT activation should be considered as a warning sign; Schneider’s research demonstrates that NIV use at the time of RRT activation is associated with a higher risk of ICU admission, underscoring the critical role of respiratory monitoring in the RRT context [46].

Although previous studies have demonstrated an association between delayed RRT activation and increased in-hospital mortality [47,48], we were unable to confirm this association in our analysis. The low median time between patient deterioration and RRT activation and between RRT activation and team arrival observed in our cohort and differences in organizational characteristics between our center and other institutions may explain, at least partially, these discrepant results.

Our study has limitations. First, the observational nature of this study and the identification of several factors associated with increased mortality do not necessarily imply causation. Second, this study was conducted in a single center, where RRT was implemented more than 15 years ago. Conversely, our findings may not apply to hospitals that have recently adopted RRT. Third, we did not investigate the impact of recurrence of RRT activation on outcomes [2,47,49]. Fourth, our study was limited to a cohort of patients admitted to the ICU/SDU following RRT activation, which precluded us from assessing patients attended by RRT but not transferred to the ICU/SDU. Finally, although respiratory distress is a well-established predictor of worse outcomes in patients following RRT activation, our results may have been affected by patients with severe acute respiratory syndrome coronavirus 2 (SARS-CoV-2) infections admitted to the UCU/SDU during the COVID-19 pandemic [50].

## Conclusion

Patients admitted to the intensive care unit or step-down unit after RRT often required organ support and exhibited an increased risk of death. Multiple factors may affect the clinical outcomes of ICU/SDU-admitted patients after RRT activation. Therefore, efforts should be made to boost RRT effectiveness to improve quality of care and patient safety.
